# Understanding the Origin and Diversity of Macrophages to Tailor Their Targeting in Solid Cancers

**DOI:** 10.3389/fimmu.2019.02215

**Published:** 2019-09-25

**Authors:** Karoline Kielbassa, Serena Vegna, Christel Ramirez, Leila Akkari

**Affiliations:** Division of Tumour Biology and Immunology, Oncode Institute, Netherlands Cancer Institute, Amsterdam, Netherlands

**Keywords:** macrophages, tumor immune microenvironment, solid tumors, immune phenotype, macrophage plasticity

## Abstract

Tumor-associated macrophages (TAMs) are a major component of the tumor immune microenvironment (TIME) and are associated with a poor prognostic factor in several cancers. TAMs promote tumor growth by facilitating immunosuppression, angiogenesis, and inflammation, and can promote tumor recurrence post-therapeutic intervention. Major TAM-targeted therapies include depletion, reprogramming, as well as disrupting the balance of macrophage recruitment and their effector functions. However, intervention-targeting macrophages have been challenging, since TAM populations are highly plastic and adaptation or resistance to these approaches often arise. Defining a roadmap of macrophage dynamics in the TIME related to tissue and tumor type could represent exploitable vulnerabilities related to their altered functions in cancer malignancy. Here, we review multiple macrophage-targeting strategies in brain, liver, and lung cancers, which all emerge in tissues rich in resident macrophages. We discuss the successes and failures of these therapeutic approaches as well as the potential of personalized macrophage-targeting treatments in combination therapies.

## Introduction

The innate immune system, which consists of macrophages, dendritic cells, neutrophils, and a variety of other effector cells, is indispensable to mount rapid defense mechanisms in the context of homeostatic disruption (1). In the late nineteenth century, Élie Metchnikoff identified macrophages and their phagocytic activity (2), and since then, these cells have been singled out as key players in innate immunity and inflammation while being essential for tissue maintenance (3).

The past decades have emphasized the importance of investigating macrophages, since they not only are responsible for tissue homeostasis but also can contribute to the pathophysiology of several diseases including development and inflammatory disorders as well as cancer, depending on their activation phenotype (4).

For many years, tissue-resident macrophages were thought to originate from bone marrow-derived progenitors and blood monocyte intermediates that differentiate into mature cells once seeded into organs (5). However, the field of development biology has recently expanded our knowledge regarding tissue macrophage ontogeny. Several genetic tracing studies revealed that multiple macrophage populations develop from embryonic progenitors and are able to self-renew by local proliferation of mature, differentiated cells (6, 7).

Each tissue microenvironment shapes macrophage morphological and functional characteristics according to the homeostatic need of its local surrounding, suggesting that macrophage tissue-specific functions are not strictly dependent on their ontogeny and that the surrounding environment imprints macrophages locally (3). Additionally, several studies reported that macrophage functions are regulated epigenetically (8, 9).

This body of work, mainly performed in the course of development and homeostasis, raises the unanswered questions of macrophage phenotype adaptation in a tissue and ontogeny-specific manner in diseased conditions.

Macrophages present in the microenvironment of tumors are referred to as tumor-associated macrophages (TAMs) and are associated with a poor prognostic factor in several solid cancers (10–12). TAMs promote tumor growth by facilitating immunosuppression, angiogenesis, and inflammation, and can also affect tumor recurrence after conventional therapies (13). These characteristics, together with their genetically stable properties, have rendered macrophages attractive therapeutic targets in the tumor immune microenvironment (TIME) (12, 14). However, the high plasticity and versatility of TAMs, their distinct embryological origins, and their evolution within the TIME during cancer progression and treatment challenge their efficient targeting.

In this review, we build a roadmap of macrophage dynamics within multiple TIME, with a particular focus on tissue and tumor specificity. We will discuss the successes and failures of macrophage targeting with relation to TAM tissue and tumor specialized functions and propose how combinatorial targeting could be implemented to utilize the yet untapped vulnerabilities of these cells in cancer.

### Macrophage Ontogeny and Education in Development and Homeostasis

Unlike most immune cells that originate from hematopoietic stem cells (HSCs), certain tissue-resident macrophages derive predominantly from embryonic progenitors, including yolk-sac macrophages and fetal liver monocytes (7, 15). The contribution of these two types of embryonic progenitors varies among different tissue-resident macrophage populations (7, 15).

The subset of resident macrophages in the central nervous system (CNS), referred to as microglia, are widely accepted to be the only tissue-resident macrophages exclusively originating from yolk sac-derived progenitors (16, 17). Embryonic microglia precursors emerge as early as E7.25 (17) and remain the sole source of macrophages in the adult brain parenchyma through their self-renewal potential.

Unlike microglia, Kupffer cells in the liver and alveolar macrophages in the lung are suggested to represent a mixed population of yolk sac macrophages and fetal liver monocytes (18). Kupffer cells and alveolar macrophages develop predominantly from erythro-myeloid progenitors (EMPs) in the yolk sac at E8.5, followed by their migration to the fetal liver by E10.5, which give rise to several cell types including fetal macrophages and monocytes (15). Fetal Kupffer cells are observed in the hepatic sinusoid at E11.0 in mouse embryos and express the macrophage marker F4/80^+^ (19), while alveolar macrophage differentiation begins with the distribution of fetal macrophages and fetal monocytes throughout the developing lung at around E12.5 and E16.5, respectively (20). Further differentiation of alveolar macrophages from fetal precursors continues until E18.5 and fully colonize the alveolar space during the first days after birth (20).

After birth, Kupffer cells and alveolar macrophages differentially rely on their potential for self-renewal and proliferation for their maintenance. While the pool of adult Kupffer cells is only marginally enriched by HSC-derived cells under steady state conditions (<5%), a substantial proportion of lung alveolar macrophages are progressively replaced during the normal aging process (7, 15).

**Figure 1:**
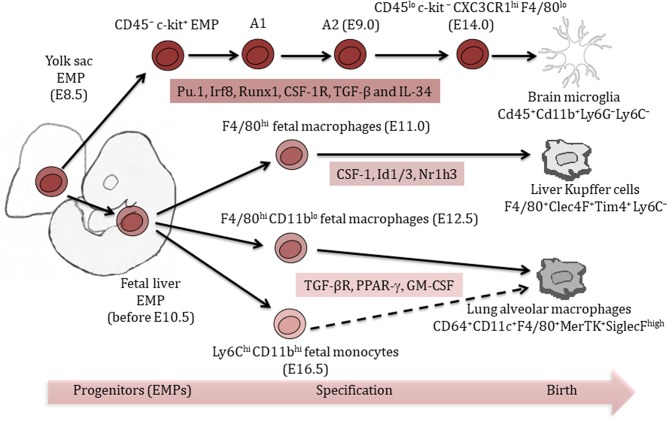
Ontogeny of tissue-resident macrophages. Liver Kupffer cells and lung alveolar macrophages originate from a mixed population of yolk sac and fetal liver erythro-myeloid progenitors (EMPs). Brain microglia exclusively arise from yolk sac EMPs. Multiple tissue-specific transcription factors are important for the differentiation and maintenance of tissue-resident macrophages. Phenotypic markers help identify different tissue-resident macrophage populations.

To differentiate and maintain their tissue-specific functions, tissue-resident macrophages rely on specific growth factors and multiple transcription factors (21). Macrophage colony-stimulating factor-1 receptor pathway (CSF-1R, ligands M-CSF/IL-34) is a crucial signaling node mediating the maintenance of Kupffer cells and microglia (22, 23), while CSF-2R/GM-CSFR appears to be essential in alveolar macrophages differentiation (24). Engagement of the transcription factors (TFs) Pu.1, Irf8, Runx1, and SMAD regulate the development of microglia (Figure 1) (17, 23, 25, 26), while myeloid TFs such as Myb, Id2, Batf3, and Klf4 are not required for microglia development (7, 17). Id2 and Id3 TFs are, however, essential to generate and maintain Langerhans cells and Kupffer cells, respectively (27, 28). Differentiation and maintenance of alveolar macrophages are dependent on transforming growth factor-β receptor (TGF-βR) signaling through the upregulation of PPAR-α (29, 30), and TGF-β is a master regulator of microglia development (Figure 1) (21, 29).

Further changes in macrophage marker expression profile occur postnatally, which distinguish them from their precursors and other tissue-resident macrophages. *Tim4* is maintained throughout development and postnatally in Kupffer cells, but lost in lung alveolar macrophages and brain microglia (28). Postnatal molecular signatures indicated tissue-specific expression of the TFs *Sall1* (26) and *Sall3* in microglia and *Nr1h3 (Lxra)* in Kupffer cells (Figure 1) (28). Molecular signatures of alveolar macrophages revealed important postnatal changes in gene expression, which might be related to their location at epithelial barriers (28). Those signatures showed for example, tissue-specific expression of the TF *Pparg* in alveolar macrophages (28).

Altogether, ample evidence supports the concept that once differentiated, each tissue-resident population of macrophages is distinct in their molecular profiles in a manner that is dependent on their embryological origin and specialized tissue education.

### Tissue-Specific Functions of Resident Macrophages

Each tissue microenvironment necessitates macrophages to undertake specific functions to maintain their physiological role in the body homeostasis. Consequently, tissue-resident macrophages adopt morphological and functional characteristics depending on their local surroundings. Unlike Kupffer cells in the liver or alveolar macrophages in the lung, microglia are the sole myeloid cells present within the healthy brain parenchyma, due to their unique ontogeny and seclusion from the peripheral circulation by the blood–brain barrier (BBB) (31). Microglia regulate the CNS homeostasis through phagocytic clearance of apoptotic neurons (32), regulation of neuronal survival, neurogenesis, and oligodendrogenesis by secreting various neurotrophic factors, including insulin-like growth factor 1 (IGF-1), IL-6, IL-1β,TNF-α, and IFN-α (33–35). Furthermore, microglia are essential in maintaining and remodeling the synaptic network (36). Synaptic pruning includes elimination of undesired synapses, which is mediated by TGF-β signaling and expression of the complement protein C3 (37).

Tissue-resident Kupffer cells mediate the tolerogenic environment of the liver and are important effectors in maintaining tissue and systemic homeostasis (38). Kupffer cells are involved in the clearance of bacteria and microbial products including pathogen-associated molecular patterns (PAMPs), damaged erythrocytes, haptoglobin–hemoglobin complexes and erythrocyte-derived hemoglobin-containing vesicles from the blood. These pleiotropic functions of liver Kupffer cells are mediated by expression of multiple Toll-like receptors (TLRs), Fc receptors, and specific scavenger receptors including SR-A (CD204), MARCO (39), and CD163 (40).

Alveolar macrophage functions are regulated by the surrounding airway epithelium through their interactions with CD200-expressing alveolar epithelial cells in the presence of TGFβ and IL-10. They are involved in surfactant lipid metabolism (41) and multiple cytokine production through the induction of PPAR-α by CSF-2 (GM-CSF) (3, 30).

Kupffer cells and alveolar macrophages have the ability to promote the suppressive activity of regulatory T cells (Tregs) by producing IL-10 (42), TGF-β, or retinoic acid (43, 44), thus leading to effector T cell immunosuppression. Alveolar macrophages additionally induce T cell antigen-specific unresponsiveness, promoting tolerance to innocuous antigens to prevent unnecessary inflammatory responses (45).

Under physiological conditions, the potential of tissue-resident macrophage self-renewal and proliferation is poorly understood. Both metabolic and nutritional signals have been suggested to regulate macrophage self-renewal (46). Similarly, the Th2 cytokine IL-4 promotes macrophage self-renewal during inflammation (47). Because these different signals are generally altered or upregulated in the course of tumorigenesis, they could favor the selective proliferation of tumor-educated, tissue-resident TAM subsets, thus fueling their pro-tumorigenic participation to the growing tumor. In that case, specific targeting of proliferative tissue-resident TAMs could represent a tantalizing therapeutic avenue to be applied early on in the course of tumorigenesis.

### TIME Shaping in Solid Tumors

The TIME consists of a large variety of immune cells with distinctive composition and functions that differ greatly between, but also within, cancer types (14, 48). Immune cells are highly heterogeneous and can exert both anti- as well as pro-tumorigenic activities depending on environmental signals they are exposed to, including inflammation and tumor cell genetic make-up.

Wide-ranging immunogenomic analysis of more than 10,000 tumors comprising 33 distinct cancer types were classified in six different immune content patterns, spanning cancer tissue types, and molecular subtypes (49). This recent study identified that several tumors, including glioma and hepatocellular carcinoma (HCC), displayed a greater range in leukocyte fraction compared to other cancer types. The immune landscape in these tumors displayed a more prominent M2-like macrophage signature, suppression of Th1 cell activation, and a high anti-inflammatory macrophage response. Lower-grade gliomas showed the lowest lymphocyte and highest macrophage responses, dominated by anti-inflammatory, M1-like macrophages (49). On the other hand, *IDH*1 mutations (also found in gliomas) correlated with low immune cell infiltration (50) and decreased leukocyte chemotaxis, resulting in fewer tumor-associated immune cells and were associated with better clinical outcome (49). Lung squamous cell carcinoma exhibited a “wound healing” immune subtype activation with elevated angiogenesis-associated genes, a high proliferation rate, and a Th2 cell bias in the adaptive immune infiltrate (49). However, these immunogenic analyses only partly integrated the tumor's genetics, and it has now been reported that deregulation of several cancer cell-intrinsic pathways influences the inflammatory TIME (48). Indeed, a few examples of how the genetic make-up of cancers affects tumor immunity have been highlighted in recent years. Expression of the tumor suppressor gene p53 together with NF-kB stimulate senescence and a senescence-associated secretory phenotype (SASP) in hepatic stellate cells, which subsequently induced a tumor-inhibiting phenotype in macrophages. Loss of p53, on the other hand, induced the activation of macrophages toward a tumor-promoting phenotype and fueled inflammation-induced HCC (48, 51). NF-kB activity was increased upon loss of p53, which promoted tumor development in the *Kras*^*LSL*−*G12D*/+^*; Trp53*^*F*/*F*^ lung adenocarcinoma model, while NF-kB inactivation resulted in increased immune cell influx and impaired lung cancer formation (52). Moreover, MYC amplification resulted in increased expression of IL-23 and CCL9 by tumor cells in lung adenocarcinoma murine models, which led to a rapid decrease of B, T, and NK cells, while macrophages were increasingly recruited and activated in the TIME (53). Myc activation has also been shown to mediate and maintain the transition from indolent pancreatic lesions (PanIN) to full adenocarcinoma (PDAC), by triggering the inflammatory ensemble of cell types characteristic of aggressive lesions. The reshaping of the PanIN TIME to a PDAC phenotype was dependent on early recruitment of macrophages through CCL9 and CCL2, and fully reversible in PDAC when Myc activity was blocked or deleted, with rapid TAM and neutrophil efflux (54). Deletion or mutations of the tumor suppressor *p53* in murine models of triple-negative breast cancer have recently been reported to enhance neutrophilic inflammation, which is mediated by tumor cell production of WNT ligands promoting the secretion of IL-1β by TAMs (55, 56). Similar results were obtained in prostate cancer models where both Ly6C+ monocyte and Ly6G+ neutrophil recruitment was blocked, giving rise to tumor growth control specifically in *pTEN* and *p53* double KO mice (57). These studies pioneered the concept of personalized immunomodulation of innate cells, in this case targeting neutrophils in *p53*-altered breast and prostate cancers. Such cancer cell genetics-guided approaches to target TAMs have not yet been explored, and could represent potent therapeutic strategies given the abundance of TAMs in multiple solid tumors. However, they also may be complicated by both TAM cell plasticity and ontogeny, highlighting the need for uncovering the dynamics of these cells in a mutational status-dependent manner.

Altogether, these reports emphasize the influence of tumor cell genetics on immune subset recruitment and activation, suggesting that tumors shape their local TIME to their advantage, which could constitute a potential vulnerability to exploit in cancer immune-modulation therapy.

### Ambivalent Role of TAM Along Tumorigenic Progression

Several studies explored the dual roles of TAMs in tumor progression of different cancer types (58). Depending on environmental stimuli, TAMs can initiate both pro-inflammatory as well as anti-inflammatory responses through their ability to directly suppress or promote the cytotoxic functions of natural killer (NK) cells and CD8+ T cells, or by triggering Th1 immune response and cytotoxic activity toward cancer cells *via* toxic intermediates production such as NO and ROS (59).

In grade IV gliomas (glioblastoma: GBM), both microglia and monocyte-derived macrophages (ontogenetically different macrophage subsets) contribute to the TAM pool and influence tumor malignancy (60). Among the non-neoplastic cells in the GBM TIME, TAMs account for 30–40% of the total GBM tumor mass, suggesting their importance in tumor maintenance and immunosuppressive features of these aggressive tumors (61). Interestingly, the density of TAMs is lower in grade II and III gliomas, in which they do not display the M2-like phenotype characteristic of grade IV tumors (62). Acquisition of this protumorigenic features in GBM relies on multiple signaling molecules promoting the M2-like polarization of TAMs, as M-CSF (CSF-1) (63).

The role of TAMs in lung cancer progression remains controversial, potentially due to different populations of macrophages analyzed in multiple tumor settings (64–67). Several reports suggest a positive correlation between TAM infiltration and favorable prognosis. Higher tumor islet densities of pro-inflammatory macrophages were associated with extended survival in non-small cell lung cancer (NSCLC), while the presence of interstitial macrophages correlated with reduced survival (66, 68). Immunostaining using CD68/iNOS (markers for pro-inflammatory macrophages) and CD68/CD163 (markers for anti-inflammatory macrophages) supported these findings with high infiltration of pro-inflammatory macrophages in the tumor islets together with low infiltration of anti-inflammatory TAMs being associated with improved NSCLC patients' survival (69). However, studies also show substantial evidence that TAMs correlate with poor prognosis in human lung cancer (70, 71). For instance, TAMs are associated with tumor IL-8 mRNA expression and increased intratumoral microvasculature, which correlates negatively with survival (70).

**Figure 2:**
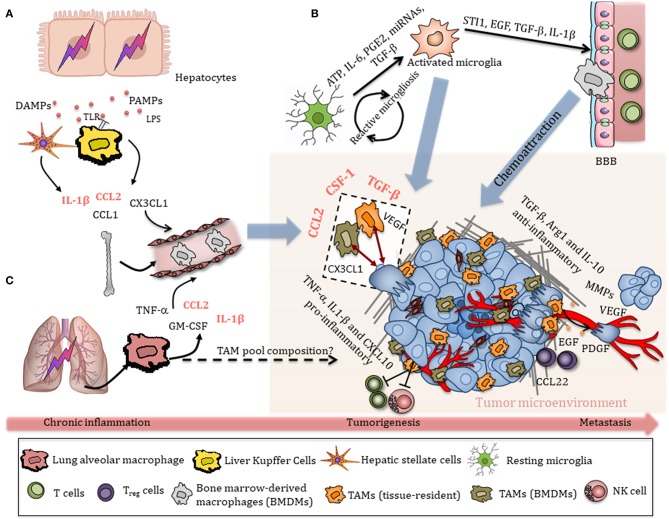
Schematic overview of TAM recruitment in the tumor immune microenvironment (TIME). **(A)** Damaged hepatocytes release a variety of DAMPs and PAMPs, which initiate an inflammatory response through activation of hepatic cells, particularly liver-resident Kupffer cells. Activated Kupffer cells, hepatocytes, and stellate cells secrete chemokines that promote the extensive recruitment of bone-marrow-derived monocytes to sites of injury. Chronic inflammation eventually contributes to tumorigenesis. TAMs are recruited in a HCC environment through CSF-1, CCL2, VEGF, and TGF-β, which in turn release many cytokines, chemokines, and growth factors that promote HCC progression. Anti-inflammatory TAMs release TGF-β, Arg1, and IL-10, as well as factors that promote tissue remodeling and angiogenesis, including VEGF, PDGF, MMP2, and MMP9. TAM-derived EGF and CCL22 recruit Treg cells, promoting metastasis. **(B)** Early tumor stimuli release various chemokines, including ATP, IL-6, PGE2, miRNAs, and TGF-β, that activate resting microglia toward an amoeboid state, which in turn modulate the BBB, allowing circulating monocytes to enter the TIME. Tumor-derived chemokines attract microglia/macrophages to the tumor, where they interact with both bulk glioma cells and glioma stem-like cells (GSCs) and contribute to tumor progression and invasiveness. **(C)** Chronic lung inflammation/injury contributes to NSCLC. Inhalation of particulate matter (PM) or cigarette smoke causes activation of alveolar macrophages *via* cell surface receptors, including TLRs, MARCO, or SR-A. Activated alveolar macrophages release a variety of pro-inflammatory cytokines, which are also released in the peripheral circulation and contribute to systemic inflammation. The relative contribution of alveolar macrophages and interstitial lung macrophages to the TAM pool and subsequently their roles in tumor progression remains unclear.

In the liver TIME, pro-inflammatory TAMs and endothelial cells produce TNF-α, which activates NF-κB and subsequently protects hepatocytes from apoptosis (72). However, exposure to IL-4, IL-10, and IL-13 triggers a switch toward an anti-inflammatory phenotype and the release of TGF-β, Arg1, and IL-10, as well as factors that promote tissue remodeling and angiogenesis, including VEGF, PDGF, MMP2, MMP9, and MT1-MMP (59, 73–75). Moreover, anti-inflammatory TAMs induce S100A8 and S100A9 expression on HCC cancer cells (76), release CCL22 and epidermal growth factor (EGF) to recruit Treg cells, and promote migration of tumor cells, altogether contributing to HCC malignancy and liver metastasis (Figure 2) (77). The role of M2-like TAMs in facilitating the epithelial–mesenchymal transition (EMT) of HCC cells in a TLR4/STAT3 signaling-dependent manner further supports the notion that these cells promote liver cancer malignancies (78).

Overall, it is now generally accepted that anti-inflammatory TAMs are mainly associated with poor survival while pro-inflammatory macrophage infiltration tends to correlate with better outcome (64–67, 70). However, limited studies have distinguished tissue-resident macrophages from monocyte-derived macrophages within solid tumors. This distinction is particularly puzzling in macrophage-rich organs like the brain, lung, and liver. In light of recent results showing that macrophage progenitors are seeded early in these organs, it is of high interest to determine the extent of resident macrophages contributing to the TAM pool and subsequently to pro- or anti-inflammatory cells, or whether they are replaced by recruited monocytes in the course of tumorigenesis.

### TAM Recruitment and Activation Within the Lung, Liver, and Brain TIME

During early tumor lesions, TAMs are recruited in the TIME through multiple secreted factors, and can in turn release several cytokines, chemokines, and growth factors that fuel tumor progression (Figure 2).

The generation of an immunosuppressive, pro-tumorigenic TIME in which macrophages are one of the most abundant immune cell players is often a consequence of chronic inflammation and organ injury (79). In the lung, inhalation of particulate matter (PM) or cigarette smoke causes activation of alveolar macrophages *via* cell surface receptors, including TLRs, MARCO, or S-RA. Activated alveolar macrophages release a variety of pro-inflammatory cytokines including TNF-α, IL-1β, IL-6, as well as IL-8 and GM-CSF (80), which promote tissue injury and recruitment of additional immune cells. Small cell lung cancer cells display high levels of monocyte chemoattractant protein-1 (MCP-1, also known as CCL2), which leads to increased recruitment of blood monocytes to tumors (Figure 2C) (81). Increased expression of IL-23 and CCL9 by tumor cells additionally promotes recruitment and activation of macrophages in the *Kras*^*G12D*^-driven lung adenocarcinoma model upon MYC amplification, as mentioned in the previous paragraph (53).

When tissue injuries cannot be resolved, chronic liver diseases such as non-alcoholic fatty liver disease (NAFLD), alcoholic liver disease (ALD), chronic HBV, or HCV infection lead to hepatic fibrosis and cirrhosis, which eventually can favor the development of hepatocellular carcinoma (HCC) (38). Resident Kupffer cells are rapidly activated by various danger-associated molecular patterns (DAMPs) or pathogen-associated molecular patterns (PAMPs) in that context, including lipopolysaccharide (LPS), viral RNA, DNA fragments, free fatty acids, uric acid, and ATP, sensed by multiple TLRs and P2X purinoreceptor 7 (P2X7), respectively (82). Activation of Kupffer cells leads to inflammasome formation and the subsequent release of various pro-inflammatory cytokines such as IL-1β, which contribute to hepatic injury (83). Kupffer cells, hepatocytes, and stellate cells also secrete chemokines, including CC-chemokine ligand 1 (CCL1), CCL2, and CX3CL1, that promote the extensive recruitment of bone-marrow-derived monocytes into the liver (38), where they differentiate into monocyte-derived macrophages (Figure 2A). During early tumor lesions, TAMs are recruited in a HCC environment mainly *via* CSF-1, CCL2, VEGF, and TGF-β, and in turn release additional cytokines, chemokines, and growth factors that promote HCC progression (59).

While high immune cell influx to sites of injury is easily achieved in the liver or in the lung, the healthy brain parenchyma is secluded from circulation by the BBB and microglia are the only myeloid cells due to their early seeding during development and specific ontogeny (31). Microglia become activated in response to early tumor stimuli, such as IL-6, TGF-β, prostaglandin E2 (PGE2), ATP, and miRNAs, which leads to the release of various cytokines, growth factors, and MMPs (84). Tumor cells, in turn, release additional mediators that will further recruit and promote another wave of microglial activation, resulting in a cyclic process in GBM, known as reactive microgliosis (Figure 2B) (84). Activated microglia switch from a resting state toward an amoeboid phenotype and subsequently release several factors that promote glioma proliferation and migration, including stress-inducible protein 1 (STI1), epidermal growth factor (EGF), TGF-β, as well as IL-1β, which modulate the BBB to further allow invasion of peripheral immune cells into the CNS (85, 86). Complementing the pool of microglia in the brain TIME, monocyte-derived macrophage recruitment is in part mediated by CSF-1, ATP, glial cell-derived neurotrophic factor (GDNF), GM-CSF, CCL2, CX3CL1, and, especially in hypoxic areas, CXCL12 (SDF-1) (61, 63, 85, 87–90). Tumor-secreted CCL2 signals through the CCR2 receptor expressed on TAMs and result in release of IL-6 to promote glioma cell invasiveness (91). In addition to bulk, differentiated glioma cells, glioma stem-like cells (GSCs) have been reported to reside in GBM perivascular niches and to be resistant to radiation and chemotherapy (92). GSCs enhance macrophage recruitment from the periphery by producing chemoattractants, such as periostin (93). Recruited TAMs can in turn influence GSCs by releasing TGF-β followed by production of MMP-9, promoting GSC invasiveness (94).

### Distinguishing Macrophage Subsets in Lung, Liver, and Brain Cancers

Macrophage populations are highly plastic and adapt their phenotype in response to microenvironmental influences (95); however, the differential responses to inflammation and tumorigenic progression in the recruited vs. resident macrophage populations is still unclear.

The challenge in distinguishing subpopulations of TAMs resides in the ability of monocyte-derived macrophages to acquire some of the tissue-resident macrophage marker expression and functionality in the course of tissue injury and tumorigenesis.

Several studies suggest that TAMs in lung tumors are largely monocyte-derived (2). During lung injury/fibrosis, resident alveolar macrophages are identified as CD64^+^CD11c^+^F4/80^+^MerTK^+^SiglecF^high^, while monocyte-derived alveolar macrophages are characterized as CD64^+^CD11c^+^F4/80^+^MerTK^+^SiglecF^low^ (96). In the *Kras*^*LSL*−*G12D*/+^*p53*^*fl*/*fl*^ lung tumor model, fluorescently labeled monocyte precursors differentiate into macrophages in developing tumors (97). Similarly, in the Lewis lung carcinoma (LLC) model of NSCLC, tracing of labeled monocyte progeny demonstrated that Ly6C^hi^ monocytes exclusively differentiate into two main TAM populations: MHC-II^lo^ anti-inflammatory and MHC-II^hi^ pro-inflammatory TAMs (98). Although both TAM subsets are derived from a common Ly6C^hi^ monocyte precursor, MHC-II^lo^ TAMs are found in hypoxic regions and upregulate hypoxia-regulated genes, such as VEGF-A, GLUT-3, GLUT-1, and iNOS, and acquire pro-angiogenic functions (98). However, a limited number of macrophage lineage tracing approaches interrogating their embryological origins have been undertaken in the context of lung tumors, thus restricting our understanding of the TAM pool composition and ontogeny-dependent functions in this disease (99).

Hepatic macrophage populations are highly plastic and adapt their phenotype in response to microenvironmental influences (95). Flow cytometry analysis identified that inflammatory monocyte-derived macrophages express high levels of Ly6C and CCR2 and are able to rapidly infiltrate tissue upon injury. Anti-inflammatory monocyte-derived macrophages, on the other hand, are Ly6C^low^, express high levels of CX3CR1, and exhibit a patrolling behavior along the liver vasculature (74). Resident Kupffer cells are F4/80^+^Clec4F^+^Tim4^+^ macrophages and negative for the marker Ly6C (15, 100). This distinction, despite being oversimplified, has been the basis for the handful of studies examining the ontogeny of macrophages in liver injury and tumorigenesis.

In injured livers, bone marrow-derived macrophages (BMDMs) were equally capable of responding to LPS and parasitic insults compared to tissue-resident Kupffer cells (KCs) (101). However, KCs were more effective at accumulating acetylated low-density lipoprotein, while BMDM uptake of a larger range of bacterial pathogens (101). KCs are the first macrophage population to respond to newly formed tumor signals and are therefore involved in HCC onset (102), while BMDMs are essential during later disease progression stages and metastasis formation (103). During HCC progression, monocyte-derived macrophages suppress the functions of effector T and B cells through expression of the immune-checkpoint molecule PD-L1 and the immunosuppressive cytokine IL-10, and are able to directly inhibit NK cells and CD8+ T cells (104). Infiltrating monocytes/macrophages lead to upregulation of S100A8 and S100A9 expression in cancer cells, which was correlated with elevated metastasis formation in HCC (76). While these recent studies shed light into the relative contribution of KC or monocyte-derived macrophages to tumor progression, the extent to which these populations are functionally and transcriptionally distinct in the course of cancer malignancy remains to be fully addressed using lineage tracing tools, as they have now been developed to examine liver macrophage homeostatic functions (100, 105).

The influence of macrophage ontogeny on brain tumor development has been the subject of thorough studies in recent years, significantly enhancing our understanding of the role of macrophage origin in this organ compared to lung or liver cancer. Traditionally, CD45 expression was used to differentiate resident microglia (MG; CD45^low^) from monocyte-derived, infiltrating BMDMs (CD45^high^) (106). Irradiation chimera experiments (where murine heads were protected from irradiation to avoid disruption of BBB) demonstrated that the main source of TAMs in primary brain tumors are resident MG, which showed an upregulation of CD45 expression in gliomas (107, 108). However, these findings were challenged by a recent study employing Cx3cr1 and Ccr2 double transgenic lineage tracing knock-in mice models, showing that recruited Cx3cr1^lo^Ccr2^hi^ monocytes differentiated into Cx3cr1^hi^Ccr2^lo^ macrophages and Cx3cr1^hi^Ccr2^−^ microglia-like cells in glioblastoma. In this study, infiltrating BMDMs represented ~85% of the total TAM population, while resident MG accounted for only the rest (109). However, using bone marrow chimera experiments and multiple lineage tracing glioma mouse models, Bowman et al. reported that MG and BMDM content was strikingly different, closer to 60 and 40%, respectively. The identification of *Itga4* (Cd49d) as a monocyte-derived macrophage-specific marker in multiple brain malignancies further confirmed these results in glioma patient samples.

While these different results may be explained by the different lineage tracing methods employed, glioma genetics or the choice of surface markers distinguishing MG and BMDMs, they all confirmed that monocyte-derived macrophages are indeed recruited to the brain TIME. Further analyses of these distinct TAM subsets revealed that glioma MG were enriched for the classical complement components C4b, C2, and pro-inflammatory cytokines such as Ccl4 and TNF-α, while BMDMs were enriched immune effectors, such as Cd40, Tlr11, Tlr5, Tlr8, Jak2, and “wound healing” chemokines, including Ccl22, Ccl17, Cxcl2, Cxcl3, and Cxcl16. These distinct signatures remain to be functionally tested and their relevance in human GBM remains to be determined. Nevertheless, the transcriptional programs specific to each macrophage subset in primary brain tumors underline their ability to distinctively respond to tumor progression.

### Monotherapies Targeting Macrophages *in vivo*

As mentioned above, numerous studies have shown substantial evidence that TAMs contribute to tumor progression and are associated with poor prognosis in solid cancers (10, 11). Major approaches targeting these cells within the TIME include macrophage depletion, macrophage reprogramming, and macrophage recruitment blockade (13, 110). However, successful macrophage-targeting strategies have been challenging to successfully implement, due to TAM high plasticity, thus giving rise to therapy resistance (110). Moreover, the vast majority of these therapeutic approaches currently target TAMs as a whole population, without fully considering their ontogeny or phenotype evolution within solid tumors.

Targeting of TAMs that have acquired protumorigenic functions presents the advantage of eliminating cells that are fueling tumor progression while preserving macrophages that may have retained their physiological, anti-tumor functions. Thus, specific depletion of M2-like TAMs is therapeutically interesting, albeit difficult to achieve. The scavenging receptor CD163, is a well-accepted marker of M2-like TAMs, and has been shown to promote their protumorigenic roles in murine and human settings (111). Genetic or nanoparticle-mediated ablation of CD163+ TAMs in melanoma leads to sustained tumor regression, partly through cytotoxic T lymphocyte recruitment and activation (112).

One of the main survival and differentiation cytokines critical to TAM biology is CSF-1, whose downstream signaling pathway can be blocked by targeting its receptor CSF-1R (113). Depending on the cancer type, blockade of CSF-1R signaling showed variable outcomes. CSF-1R blockade using the receptor tyrosine kinase (RTK) small drug inhibitor BLZ945 (Novartis) limits glioma progression and leads to regression of established tumors (114). Mechanistically, CSF-1R inhibition mediated the reprogramming of TAMs toward an anti-tumorigenic phenotype, without depleting cells within the tumor bulk (114, 115). However, long-term exposure to CSF-1R inhibition as a monotherapy was found associated with PI3K hyper-activation driven by IGF-1 production in the TIME (115). Although the CSF-1R inhibitor PLX3397 showed anti-tumor efficacy in a pre-clinical glioma model (63), these findings were not translated in recurrent glioblastoma patients (116). Human glioblastoma frequently bear alterations of PI3K and PTEN (117), which might be associated with inherent resistance to CSF-1R targeting, readily explaining the results of this clinical trial. These results underline the importance of identifying the acquired resistance to long-term macrophage targeting and consequently adapt treatment in a personalized manner, similarly to what has been done for targeted therapies (110).

The effect of CSF-1R targeting was strikingly different in breast and in cervical pre-clinical murine models. CSF-1R inhibition led to macrophage depletion, thereby causing increased infiltration of CD8^+^ cytotoxic T cells and improving responses to chemotherapy and radiotherapy (10, 118, 119). Thus, CSF1-R targeting leads to different consequences in the TIME, causing either depletion or macrophage reprogramming in a tumor-specific manner. It is yet to be determined whether blockade of CSF-1R affects predominantly tissue-resident or recruited macrophages in solid tumors. In inflammatory models however, CSF-1R blockade mediated depletion of tissue-resident macrophages, which resulted in enhanced recruitment of pro-inflammatory monocytes. These results support the hypothesis that continuous CSF-1R inhibition would then be needed to behold therapeutic effects, as tissue-resident cell depletion could be compensated by recruitment of macrophage progenitors to replenish the pool of tissue/tumor-associated macrophages (120).

Another approach to limiting macrophage pro-tumorigenic roles in the TIME is to prevent their recruitment by inhibiting the chemokine gradients' axes they rely on, including Cxcl12 (SDF-1)/Cxcr4. Blocking Cxcr4 using the inhibitor AMD3100 resulted in reduced metastatic properties of mammary tumors (121). In metastatic melanoma, Cxcl12 favors monocyte differentiation into perivascular macrophages, thus enabling the establishment of an autocrine CXL12/CXCR4 loop promoting further leucocyte infiltration and metastatic progression (122). Tumor cell-secreted CCL2 also acts as a monocyte-attracting chemokine to recruit myeloid cells in several metastatic niches (123). Blockade of the CCL2/CCR2 axis led to reduction of monocyte infiltration in multiple TIME (124) and inhibits breast cancer cell metastatic seeding (123, 125). However, cessation of this CCL2/CCR2 blockade can lead to compensatory phenotype associated with increased breast cancer metastasis, for instance (126).

Several clinical trials are currently testing CSF-1/CSF-1R targeting agents (including the Novartis small-molecule inhibitor BLZ945 and Roche monoclonal antibody RG7155) in, among others, breast cancer, glioma, melanoma, ovarian cancer, and lung cancer (Table 1) (132). So far, only RG7155 has yielded therapeutically beneficial outcomes as a single agent in diffuse-type giant cell tumors patients and has been shown to deplete CSF-1R^+^CD163^+^ macrophages (113). CCR2 inhibitors (MLN1202 and PF-04136309) are utilized for their ability to reduce bone marrow-derived cell recruitment in metastatic cancers and as first-line treatment in pancreatic tumors (Table 1).

**Table 1:** Summary of recent clinical trials using macrophage-targeting therapies.

**Company**	**Drug**	**Targets**	**Phase**	**References**
**MONOTHERAPY**				
Novartis	BLZ945	CSF-1R *Advanced* *Solid tumors*	I/II	*In progress* NCT02829723
Roche	RG7155	CSF-1R *Locally advanced and/or metastatic ovarian and breast carcinoma*	I	*In progress* NCT01494688
Southwest oncology group	MLN1202	CCR2 *Bone metastasis*	I/II	*In progress* NCT01015560
Pfizer	PF-04136309	CCR2 *First-line metastatic pancreatic cancers*	I/II	*Terminated* NCT02732938
	Sorafenib	VEGFR, PDGFR	III	(127)
Bayer	Regorafenib	VEGFR1-3,PDGFRβ, FGFR *Hepatocellular carcinoma*	III	(128)
Centocor	CNTO888	CCL2 *Castration-resistant prostate cancer*	II	(129)
**COMBINATION WITH IMMUNOTHERAPY**				
	CP870893 + taxol/carboplatin	CD40 *Metastatic melanoma*	I	(130)
	BLZ945 + PRD001	CSF-1 PD-1 *Advanced solid tumors*	I/II	*In progress* NCT02829723
	LY3022855 + Tremelimumab	CSF-1R CTLA-4 *Advanced solid tumors*	I	*In progress* NCT02718911
***COMBINATION WITH CHEMOTHERAPY***				
Plexxicon	PLX3397 + Temozolomide	CSF1R, KIT, FLT3 *Advanced solid tumors*	I/II	*In progress* NCT01525602
Pfizer	PF-04136309 FOLFIRINOX	CCR2 *Advanced pancreatic adenocarcinoma*	I/II	*In progress* NCT01413022
Centocor	CNTO888+ Docetaxel Gemcitabine Paclitaxel+Carboplatin Doxorubicin	CCL2 *Advanced solid tumors*	II	(131)
**COMBINATION WITH RADIOTHERAPY**				
Plexxicon	PLX-3397	CSF1R, KIT, FLT3 *Primary glioblastoma*	I/II	*In progress* NCT01790503

### Combination (Pre-) Clinical Therapies of Macrophage Targeting With Cytotoxic and Immunotherapy Agents

It is only in recent years that strategies using macrophage-targeting agents have been combined with targeted therapies, standard of care treatment or immunotherapies, revealing the potential of these approaches in solid tumors.

Combination of CSF-1R targeting with other RTK inhibitors showed enhanced treatment outcomes in several studies (133, 134). PLX3397 combined with the mTOR inhibitor rapamycin inhibits outgrowth of malignant peripheral nerve sheath tumors when compared to single drug treatment (133). In glioma, when combined with the multi-targeted kinase inhibitors vatalanib and dovitinib, PLX3397 did not induce depletion but depolarization of pro-tumorigenic TAMs and resulted in pronounced glioma regression (135). Moreover, both TAM reprogramming and depletion of TIE2-expressing macrophages (TEMs) together with VEGF ligand inhibition showed anti-tumor effect in orthotopic glioma models (136). In pre-clinical melanoma models, treatment with CSF-1R inhibitors enhanced the efficacy of the BRAF inhibitor vemurafenib, which was associated with depleted macrophages, allowing increased anti-tumorigenic CD8^+^ cytotoxic T cell infiltration (134).

The efficacy of CSF-1R inhibitors when concurrently administered with chemotherapy has also been reported. TAMs suppress the cytotoxic activity of antimitotic agents, including taxol, in breast cancer and promote early mitotic slippage (137). Mechanistically, TAM depletion favors increased levels of cancer cell-DNA damage and cell death in response to taxol, thus enhancing the response to this chemotherapy (137). Moreover, the combination of PLX3397 with the chemotherapeutic drug temozolomide and radiotherapy (NCT01790503) are currently assessed in phase 1b/2 study (18). Immune-cold tumors, such as pancreatic ductal adenocarcinoma (PDA), lack robust T cell infiltrates and are resistant to chemotherapy (138). Targeting TAMs in these types of tumors have thus been tested in order to lessen immunosuppression. Stimulation of the CD40 receptor at the cell surface of macrophages using agonistic CD40 antibody promotes clonal T cell expansion and combination treatment with chemotherapy resulted in optimized myeloid activation and T cell function (138). These results led to the development of a phase I study in which the agonistic CD40 antibody CP870893 (Pfizer) was combined with carboplatin and Taxol, confirming the safety of this treatment regimen (139). Clinical trials in which CSF1R inhibitors are combined with immunotherapy are currently ongoing, such as BLZ945 with PRD001 [monoclonal antibody targeting programmed cell death-1 (PD1)] and LY3022855 with tremelimumab [monoclonal antibody targeting cytotoxic T-lymphocyte-associated protein 4 (CTLA-4)] in advanced solid tumors (140).

Components of the TIME have pivotal roles in determining treatment outcomes of radiotherapy (RT) (141). RT leads to increased T cell recruitment to the TIME and can prime the immune system against cancer cells *via* immunogenic cell death (ICD). However, enhanced actions of suppressive immune cells such as TAMs constrain the efficacy of RT. TAMs are highly radioresistant and produce increased levels of Arg-1, COX-2, and iNOS post-irradiation, which promote early tumor regrowth (142). Meng et al. administered clodronate liposomes systemically or locally before RT, to deplete circulating and resident TAMs, which increased the anti-tumorigenic effects of ionizing radiation (143). Inhibition of CSF-1R using PLX3397 delays recurrence of GBM after ionizing radiation by altering myeloid cell recruitment and polarization (144). RT also causes increased TNF-α-induced VEGF ligand production by TAMs and VEGF-neutralizing antibodies enhanced the anti-tumor efficacy to RT (143). In PDAC, RT leads to the production of CCL2, which recruits macrophages to tumor sites to support tumor proliferation and neo-angiogenesis after RT. Therefore, disrupting the CCL2–CCR2 axis in combination with RT may improve RT efficacy in PDAC (145).

### Personalized Approaches Targeting Macrophages

Personalized macrophage targeting, tailored-based on tissue and tumor types, could represent a significant advance in the development of effective and long-lasting treatments. However, as mentioned in the previous section, more knowledge is needed to take on these approaches, and multiple tumor cell-intrinsic and extrinsic factors should be considered with regard to TAM immunomodulation (146).

High-throughput analysis of GBM samples shows that many types of mutations, including mutations to TP53, PTEN, or NF-1, occur in gliomas, which may profoundly affect tumor–host interactions (117, 147). Different driver mutations can co-exist; therefore, targeting of single activated pathways has led to unsuccessful therapeutic outcomes in GBM. Gain-of-function mutations of TP53 promote inflammation in GBM through upregulation of CCL2 and TNF-α expression via NFκB signaling, which consequently lead to increased infiltration of microglia and monocyte-derived immune cells in the TIME (148). NF1 loss in glioblastoma is also associated with increased macrophage infiltration displaying pro-tumorigenic features (149). Because TP53 and NF1 mutations are characteristic of the mesenchymal and proneural subtypes, respectively, it would be important to select specifically these patients in clinical trials targeting TAMs. Loss of PTEN induces increased expression of PD-L1, which correlates with PI3K expression and immune escape in GBM (150). Therefore, including PD-1/PD-L1 pathway inhibition in PTEN-mutated GBM, together with TAM targeting, could be an efficient treatment strategy, as suggested by the combinatorial effects of PI3K and CSF-1R inhibitors to reprogram macrophages toward an anti-tumorigenic phenotype (110). The recent reports identifying either a dominance of microglia or infiltrating macrophages in treatment-naïve gliomas may be due to the distinct genetic make-up of these tumors, as suggested above, and TAMs adopt distinct programming dependent on their ontogeny. Specific targeting of one or the other subsets of TAMs may thus prove to be advantageous to alleviate the protumorigenic functions of the targeted populations while maintaining the homeostatic functions of the other.

Non-small cell lung cancer (NSCLC) is characterized by driver mutations, including epidermal growth factor receptor (EGFR), anaplastic lymphoma kinase (ALK), and KRAS, permitting the efficacy of targeted therapies [reviewed in (151)]. KRAS mutations, however, are not targetable. The immune landscape of these tumors shows increased intratumoral myeloid and T cells (152), while loss of LKB1 in KRAS-mutated NSCLC results in higher levels of CXCL7, G-CSF, and IL-6, which promote neutrophil recruitment and macrophage activation and thereby suppress T cell activity (153). In these tumors, targeting the LKB1/AMPK pathway by activating AMPK may control tumor growth through limiting myeloid cell infiltration and polarization (154). In lung tumors not harboring targetable oncogenic mutations, T cell immunotherapy has yielded partial success. These could potentially be improved by targeting myeloid cell tissue remodeling and immunosuppressive functions to enhance the efficacy of T cell immunotherapy. The association between major driver mutations in lung cancer and PD-L1 expression on myeloid cells remains debated and EGFR mutated lung tumors have been reported to display both low (155) and high (156) PD-L1 expression. Therefore, a better understanding of PD-L1 expression in specific subsets of TAMs would be useful to target these cells specifically.

The role of tissue-resident macrophages has been well-established in clearing pre-cancerous hepatocytes in the liver (157), and senescence in the liver environment can promote the anti-tumor properties of TAMs in early stages of neoplasia (51). NOTCH signaling amplification limits the anti-tumorigenic response mediated by oncogene-induced senescence via the secretion of the senescence-associated pro-inflammatory cytokines IL-1β, IL-6, and IL-8 (158). Accordingly, targeting amplified NOTCH signaling could increase anti-tumorigenic efficacy through promoting senescence surveillance by myeloid cells. Mutated NRAS in liver cancer results in increased recruitment of anti-tumorigenic TAMs through the senescence-associated secretory phenotype (SASP) and promotes CD4^+^ T cell-mediated clearance of liver pre-neoplastic cells (159). Similarly, amplification of mTOR in liver cancer leads to increased levels of IL-1β, which activates NF-kB, thereby driving tumor suppressive SASP and immune cell recruitment (160). Furthermore, loss of AKT in liver cancer results in decreased content of pro-tumorigenic Wnt-producing macrophages and thereby limits tumorigenesis (161). Therefore, Wnt/β-catenin targeting may inhibit tumorigenic activities of macrophages (161). Altogether, these studies suggest that generating senescence in established HCC and modulating the SASP may represent a potent approach to reprogram TAMs in liver cancer, which would need to be tailored to the type of senescence generated in the liver TIME (159).

## Discussion

Targeting different subsets of macrophages instead of pan-macrophages could improve disease outcomes by hampering the pro-tumorigenic functions of specific subsets of TAMs and protecting the homeostatic properties of others (114, 136). However, this requires understanding and considering multiple features of these cells such as the following: identifying the adequate surface markers for distinguishing different macrophage subsets in specific organs, deciphering their recruitment and activation dynamics in the course of tumor progression and response to therapy, and defining the shaping they are conditioned to by the genetic make-ups of tumors. In this review, we discussed tissue-specific functions of resident macrophages under homeostatic conditions and in malignancy. We propose that tissue-resident macrophage populations should be targeted during tumor initiation, since they are often involved in early inflammatory processes and are a major contributor to the recruitment of monocyte-derived macrophages. Meanwhile, monocyte-derived macrophage subsets may be best targeted at later time points of tumor progression, since they are often involved in tumor invasiveness and immunosuppression. However, considerable work should be undertaken to better understand the contribution of TAM origin to tumor progression, which requires employing lineage-tracing studies in the context of chronically inflamed tumors in particular. The majority of macrophage-targeting approaches are focusing on CSF-1R inhibition to either deplete or reprogram macrophage populations toward an anti-tumorigenic phenotype and several studies are focusing on combining chemotherapy and immunotherapy with CSF-1R inhibitors (10, 118). However, it is still unclear whether inhibition of CSF-1R similarly affects monocyte-derived and tissue-resident macrophage subsets in different cancer types, which needs to be addressed to fully capture either the efficacy or failures of such treatments.

Importantly, the use of sophisticated mouse models closely reproducing the human genetics of tumors and composition of the TAM pool will be essential to test the efficacy and long-lasting effects of macrophage-centric therapies, as potential resistance could emerge.

Overall, we are only beginning to appreciate the potential of macrophage subset reprogramming. Rather than depleting them, re-educating TAMs into a homeostatic activation state and controlling the recruitment of immunosuppressive subsets could boost anti-tumor immunity. These novel therapeutic avenues could then hold promise for the development of effective anti-cancer treatments, particularly when used synergistically with tumor- or T cell-centric therapies.

## Author Contributions

KK, SV, and LA conceptualized the study. KK, LA, SV, and CR wrote the manuscript.

### Conflict of Interest

The authors declare that the research was conducted in the absence of any commercial or financial relationships that could be construed as a potential conflict of interest.
